# Food Reservoir for *Escherichia coli* Causing Urinary Tract Infections

**DOI:** 10.3201/eid1606.100158

**Published:** 2010-06

**Authors:** Maria Giufrè, Caterina Graziani, Marisa Accogli, Marina Cerquetti

**Affiliations:** Istituto Superiore di Sanità, Rome, Italy

**Keywords:** Escherichia coli, urinary tract infections/microbiology, genotype, food, bacteria, letter

**To the Editor:** We read with interest the article by Vincent et al. that compared *Escherichia coli* isolates from 3 sources (human urinary tract infections [UTIs], retail meat, and restaurant/ready-to-eat foods) by multiple molecular typing methods (*1*). This study has to be considered in the context of the larger debate about the possible animal origin of *E. coli* isolates that cause extraintestinal infections in humans (*2*–*5*), and the same authors (Vincent et al.) have declared, in the introduction, that their efforts were directed toward investigating the hypothesis that retail chicken is the main reservoir for extraintestinal *E. coli*.

We strongly appreciate the amount of the experimental data and some interesting findings, but we are not totally convinced of the authors’ conclusions, particularly the assumption that the study strongly supports the preliminary hypothesis. First, the observation that only a low proportion (73/844, 8.6%) of the *E. coli* isolates analyzed belonged to clonal groups (defined as >2 *E. coli* isolates that had indistinguishable multilocus variable number tandem repeats and enterobacterial repetitive intergenic consensus 2 patterns), including members from >1 source, suggests an overall high degree of genetic heterogeneity among isolates from different sources. Second, looking at the single isolates within clonal groups reported in, twelve (2.9%) of the 417 isolates from retail meat shared multilocus variable number tandem repeats, enterobacterial repetitive intergenic consensus 2, and multilocus sequence types with some human UTI isolates; however, only 1 isolate (strain EC01DT06–1737–01) was also found to be indistinguishable from a human isolate (strain MSHS 161) by pulsed-field gel electrophoresis , indicating that identical genotypes (between isolates from retail meat and human infections) were observed only once.

Although we agree that the finding of a partial overlap between multilocus sequence types of isolates from retail meat and from human UTI isolates is noteworthy (especially recovery of an ST131 isolate of avian origin), the emphasis posed for the role of food transmission in the dissemination of the *E. coli* strains that cause community-acquired UTIs, in our opinion, does not seem strongly supported by the experimental data. Nevertheless, the topic is relevant, and we would highlight the importance of further research on this issue.
