# Supplementary material for: Food Reservoir for Escherichia coli Causing Urinary Tract Infections
Source: Emerg Infect Dis. 2010 Jun;16(6):1048–9. doi: 10.3201/eid1606.100158 (PMC3086230; doi:10.3201/eid1606.100158)
Supplement: Related texts

# Letter: Food Reservoir for *Escherichia coli* Causing Urinary Tract Infections

**DOI:** 10.3201/eid1606.100407

**Published:** 2010-06

**Authors:** Amee R. Manges

**Affiliations:** McGill University, Montreal, Quebec, Canada

**In Response:** Giufrè et al. (*1*) responded to our recent article about the possibility of a food reservoir, specifically in retail chicken meat, for *Escherichia coli* causing human extraintestinal infections (*2*). They are not convinced by the data of “strong support” for the hypothesis that retail chicken meat could be a reservoir for these *E. coli* organisms and indicate that the observed proportion of total clonal group members (8.6%) is low and heterogeneity is significant among the isolates tested.

We assembled the 844 study isolates from 3 sources (human, retail meat, and restaurant-ready-to-eat foods) in 2 provinces during a 3-year period. Given the ecologic design of the study, the fact that 72 isolates actually were related across these sources is surprising and compelling. Furthermore, we identified a retail chicken meat isolate and human urinary tract infection isolate that were indistinguishable by pulsed-field gel electrophoresis (PFGE), again a surprising result given the study design. PFGE remains the standard for *E. coli* genotyping because of its discriminatory power; these results were also confirmed by PFGE by using a second enzyme. This group was identified as containing *E. coli* O25:H4–ST131, a clonal group that appears to cause extraintestinal disease worldwide.

Our study is among the first to extensively genotype *E. coli* isolates from these sources. Hence, the amount of genetic diversity expected in these *E. coli* organisms is unclear. This diversity is the primary issue raised by Giufrè et al.: how much genetic relatedness would be expected in a comparison of *E. coli* isolates from these sources? Although our study was fairly modest in size and was limited by the study design, we observed evidence supporting our primary hypothesis that retail chicken meat may be a reservoir for *E. coli* causing extraintestinal infections in humans. More studies certainly will help resolve the debate.

## References

[1] Giufrè M, Graziani C, Accogli M, Cerquetti M. Food reservoir for *Escherichia coli* causing urinary tract infections [letter]. Emerg Infect Dis. 2010;16:1048–9.2050777910.3201/eid1606.100158PMC3086230

[2] Vincent C, Boerlin P, Daignault D, Dozois CM, Dutil L, Galanakis C, Food reservoir for *Escherichia coli* causing urinary tract infections. Emerg Infect Dis. 2010;16:88–95. 10.3201/eid1601.09111820031048PMC2874376
